# Identification of differentially expressed genes and signaling pathways in chronic obstructive pulmonary disease via bioinformatic analysis

**DOI:** 10.1002/2211-5463.12719

**Published:** 2019-09-29

**Authors:** Xinwei Huang, Yunwei Li, Xiaoran Guo, Zongxin Zhu, Xiangyang Kong, Fubing Yu, Qiang Wang

**Affiliations:** ^1^ Faculty of Environmental Science and Engineering Kunming University of Science and Technology China; ^2^ Medical School Kunming University of Science and Technology China; ^3^ Department of Pharmacy Kunming Children's Hospital China; ^4^ Department of Gastroenterology Fourth Affiliated Hospital of Kunming Medical University China; ^5^ Physical Examination Center Second People's Hospital of Yunnan Province Kunming China

**Keywords:** bioinformatic analysis, chronic obstructive pulmonary disease, differentially expressed gene, epidemiology, GEO data

## Abstract

Chronic obstructive pulmonary disease (COPD) is a multifactorial and heterogeneous disease that creates public health challenges worldwide. The underlying molecular mechanisms of COPD are not entirely clear. In this study, we aimed to identify the critical genes and potential molecular mechanisms of COPD by bioinformatic analysis. The gene expression profiles of lung tissues of COPD cases and healthy control subjects were obtained from the Gene Expression Omnibus. Differentially expressed genes were analyzed by integration with annotations from Gene Ontology and Kyoto Encyclopedia of Genes and Genomes, followed by construction of a protein‐protein interaction network and weighted gene coexpression analysis. We identified 139 differentially expressed genes associated with the progression of COPD, among which 14 Hub genes were identified and found to be enriched in certain categories, including immune and inflammatory response, response to lipopolysaccharide and receptor for advanced glycation end products binding; in addition, these Hub genes are involved in multiple signaling pathways, particularly hematopoietic cell lineage and cytokine‐cytokine receptor interaction. The 14 Hub genes were positively or negatively associated with COPD by wgcna analysis. The genes *CX3CR1*,*PTGS2*,*FPR1*,*FPR2*,* S100A12*,*EGR1*,*CD163*,* S100A8* and *S100A9* were identified to mediate inflammation and injury of the lung, and play critical roles in the pathogenesis of COPD. These findings improve our understanding of the underlying molecular mechanisms of COPD.

**Abbreviations**
ARG1: arginase 1
BP: biological process
CC: cellular component
COPD: chronic obstructive pulmonary disease
CS: cigarette smoke
CX3CR1: C‐X3‐C motif chemokine receptor 1
DALY: disability‐adjusted life year
DEG: differentially expressed gene
EGR1: early growth response 1
FC: fold change
FGG: fibrinogen gamma chain
FPR1: formyl peptide receptor 1
FPR2: formyl peptide receptor 2
GO: Gene Ontology
GS: gene significance
KEGG: Kyoto Encyclopedia of Genes and Genomes
MF: molecular function
MM: module membership
ORM1: orosomucoid 1
PPBP: proplatelet basic protein
PPI: protein‐protein interaction
PTGS2: prostaglandin‐endoperoxide synthase 2
S100A12: S100 calcium binding protein A12
S100A8: S100 calcium binding protein A8
S100A9: S100 calcium binding protein A9
VCAM1: vascular cell adhesion molecule 1
wgcna: weighted gene coexpression network analysis
YLLs: years of life lost

Chronic obstructive pulmonary disease (COPD), characterized by long‐term poorly reversible airway limitation and persistent respiratory symptoms, is a common and preventable disease 1. COPD is projected to become the third leading cause of all death by 2030 in the world 2. Globally, COPD affected 299.4 million people in 2017, with a 71.2% increase in the prevalence rate compared with 2015, ranking it as the fifth leading cause of disability‐adjusted life years (DALYs) and the seventh leading noncommunicable disease cause of years of life lost (YLLs) 3, 4, 5, 6. As shown in Fig. 1A, we observed a 12.3% increase in global all‐age deaths caused by COPD from 2.85 million in 1990 to 3.20 million in 2017 6, 7, 8, 9 and a predicted increase of 60% by 2020 compared with 1990. Figure 1B indicated that the all‐age standardized death rate of COPD in males, females, and both sexes separately decreased from 1990 to 2015 6, 8, which could be because of population growth and aging. Although the COPD death rate varies with different countries, more than 90% of COPD deaths occurred in low‐ and middle‐income countries 10. The global all‐age YLLs with COPD showed a small increase of 7.5% and 3.6% for both sexes and males, respectively, as well as a 21% decrease for females from 1990 to 2015 (Fig. 1C) 6, 8. In addition, as shown in Fig. 1D 3, 4, 5, 7, 11, 12, 13, 14, global all‐age DALYs caused by COPD had a small increase of 4.2% during 1990–2015 and was projected to decline to 57.6 million by 2020. The age‐standardized DALY rate caused by COPD in females was about twice as high as that of males, and that in low‐ and middle‐income countries was 6.7 times higher than in some high‐income countries 3. We observed that the global all‐age years lived with disability caused by COPD has grown 52.2% from 1990 to 2017. Taken together, COPD has presented a global public health challenge with high prevalence, mortality and disability rates, whereas the diagnosis of COPD is usually made based on spirometry values and clinical symptoms with a frequent underdiagnosis 15. Thus, it is important to explore the underlying molecular mechanisms and identify more effective early diagnostic methods and reliable biomarkers for this disease.

**Figure 1:**
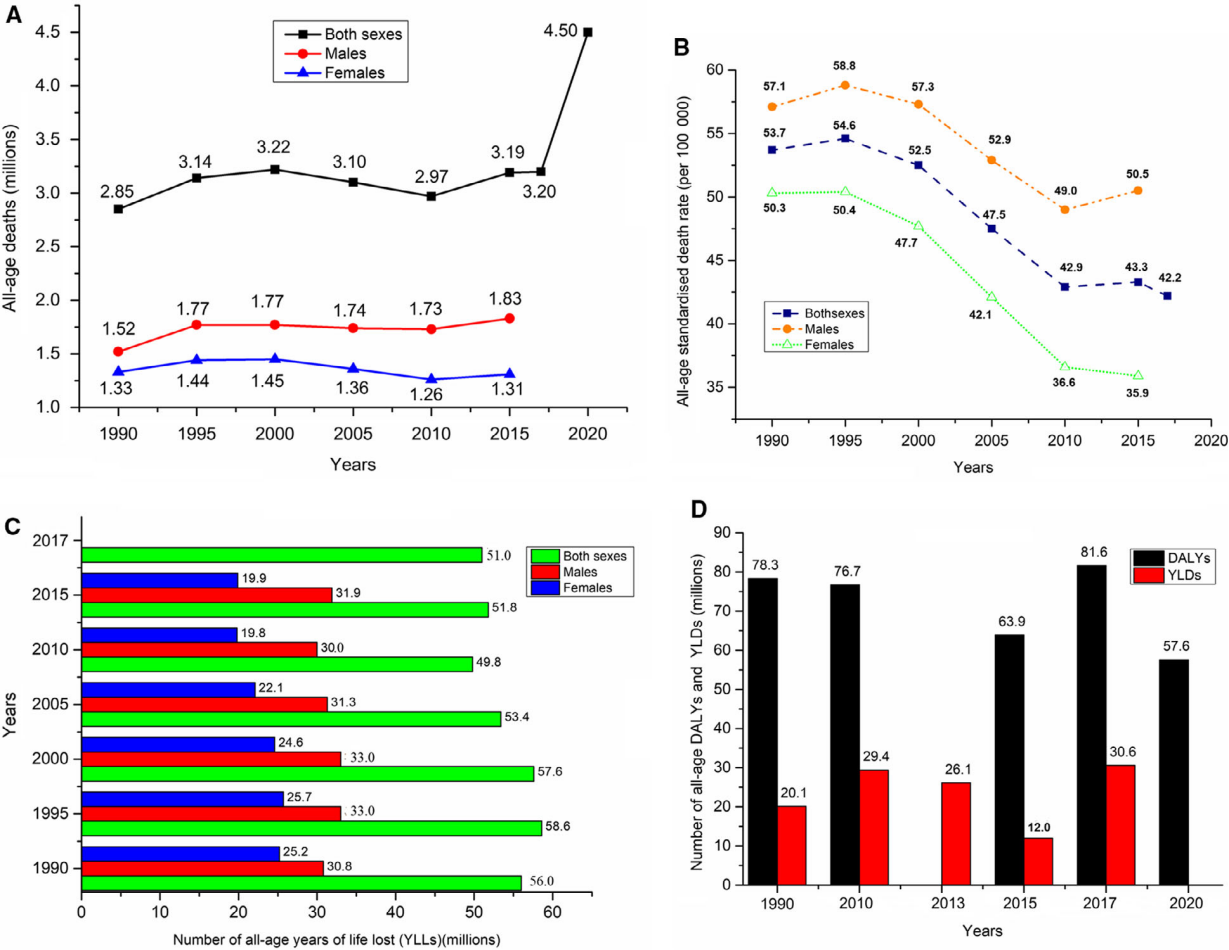
The global death and burden caused by COPD. (A) Global age‐related deaths (millions) caused by COPD in men and women, respectively, from 1990 to 2020 6, 7, 8, 9. (B) Global age‐related death rates (per 100 000) caused by COPD for both sexes, males and females, respectively, from 1990 to 2015 6, 8. (C) Global age‐related YLLs (millions) caused by COPD for both sexes, males and female, respectively, from 1990 to 2015 6, 8. (D) Global age‐related DALYs and years lived with disability (YLD) (millions) by COPD for both sexes from 1990 to 2020 3, 4, 5, 7, 11, 12, 13, 14.

As a large‐scale and efficient technique for acquiring genomic data, microarray‐based gene expression profiles have been widely used to seek new insights for biomarkers in many human diseases 16. Currently, many studies have been conducted on COPD gene expression profiles, and these studies have screened thousands of differentially expressed genes (DEGs) implicated in the development and progression of this disease 17, 18. However, the results for the identification of DEGs are discrepant among these studies due to sample heterogeneity and differences in technological detection platforms. In this study, we performed an integrated analysis on some of the gene expression profiling data based on lung tissues of COPD cases and control subjects using an unbiased approach aiming to identify the potential molecular mechanisms and biomarkers for COPD.

We selected two Gene Expression Omnibus microarray datasets on COPD ( GSE27597 and GSE106986). DEGs were identified by r software (Auckland, New Zealand) and subsequently analyzed using bioinformatic methods including Gene Ontology (GO) and Kyoto Encyclopedia of Genes and Genomes (KEGG) pathway enrichments and construction of protein‐protein interaction (PPI) and weighted gene coexpression analysis (wgcna) networks. We screened the DEGs for potential association with the development and progression of COPD. Our work may further the understanding of the potential molecular mechanisms of COPD.

## Materials and methods

### Gene data

Two gene expression datasets, GSE27597 and GSE106986, were downloaded from the National Center for Biotechnology Information Gene Expression Omnibus database (https://www.ncbi.nlm.nih.gov/geo/). GSE27597 comprises the expression profile from 64 lung tissue samples from COPD cases and 16 samples from healthy donors 19. GSE106986 includes molecular profiling of 19 lung tissue samples, containing 14 samples from COPD cases and 5 from smokers 20. The experiments of GSE27597 and GSE106986 were conducted on the Affymetrix Human Exon 1.0 ST GeneChip (Affymetrix, Inc., Santa Clara, CA, USA; GPL5175 platform) and Agilent‐026652 Whole Human Genome Microarray 4x44K v2 (Agilent Technologies, Inc., Palo Alto, CA, USA; GPL13497 platform), respectively.#AuthorQuery Rep

### Data preprocessing and screening for DEGs

The probe set IDs of two datasets were converted into gene symbols using the r software and annotation packages. The two datasets were merged into one array dataset and then batch normalized using r packages (sva and limma 3.40.6). The DEGs between COPD cases and control subjects were identified using the limma package in r 3.60. A *P*‐value <0.05 after being adjusted by false discovery rate and |log2FC > 1, where FC represents fold change, were applied together as the cutoff for DEGs screening.

### GO and KEGG pathway enrichment analysis of DEGs

GO enrichment of the DEGs on the biological process (BP), molecular function (MF) and cellular component (CC) categories was performed using a david online tool (https://david.ncifcrf.gov/) 21, 22. KEGG pathway enrichment analysis was performed by using the KOBAS 3.0 online analysis database (http://kobas.cbi.pku.edu.cn/) 23.

### Construction of the PPI network

STRING database (https://string-db.org/) is frequently used for identifying the protein interactions 16, 24. STRING database contains huge amounts of experimental and text mining data 25. cytoscape is an open source bioinformatic software platform used for integrating gene expression profiles and visualizing molecular interaction networks. cytoscape plugin cytoHubba provides multiple topological analysis methods on Hub genes, regulatory networks and drug targets for experimental biologists 26. In this study, we used STRING database to identify the interactions between the identified DEGs. A confidence score >0.4 was used as the cutoff criterion. In addition, Hub DEGs were identified with cytoscape version 3.6.1 (1999 Free Software Foundation, Inc., Boston, MA, USA) with cytoHubba plugin according to the rank of connection degree (number) for each gene, which is represented by the different degrees of color (from red to yellow): the role of the gene is greater in the PPI network with the darker color of the gene 26.

### wgcna on COPD

wgcna may be used for screening modules (clusters) of highly correlated genes and for calculating module membership (MM) measures, in which the module eigengene or an intramodular Hub gene is used to summarize such modules, and eigengene network methodology is used for relating modules to one another and to external sample traits 27. In this study, the wgcna package was used to identify coexpression modules for the merged and standardized array datasets (GSE27597 and GSE106986). In brief, first, a weighted adjacency matrix containing pairwise connection strengths was constructed based on the selected soft threshold power (β = 11) on the matrix of pairwise correlation coefficients. Then, the connectivity measure per gene was calculated by summing the connection strengths with other genes; modules were defined as branches of a hierarchical clustering tree by using a dissimilarity measure, and each module was assigned a color. Subsequently, module preservation r function was used to assess the module structure preservation. Finally, the module eigengene was used for summarizing the gene expression profiles of each module, and each module eigengene was regressed on case trait (COPD) and smoking status by using the linear model in the limma r package.

## Results

### Identification of DEGs in COPD

**Figure 2:**
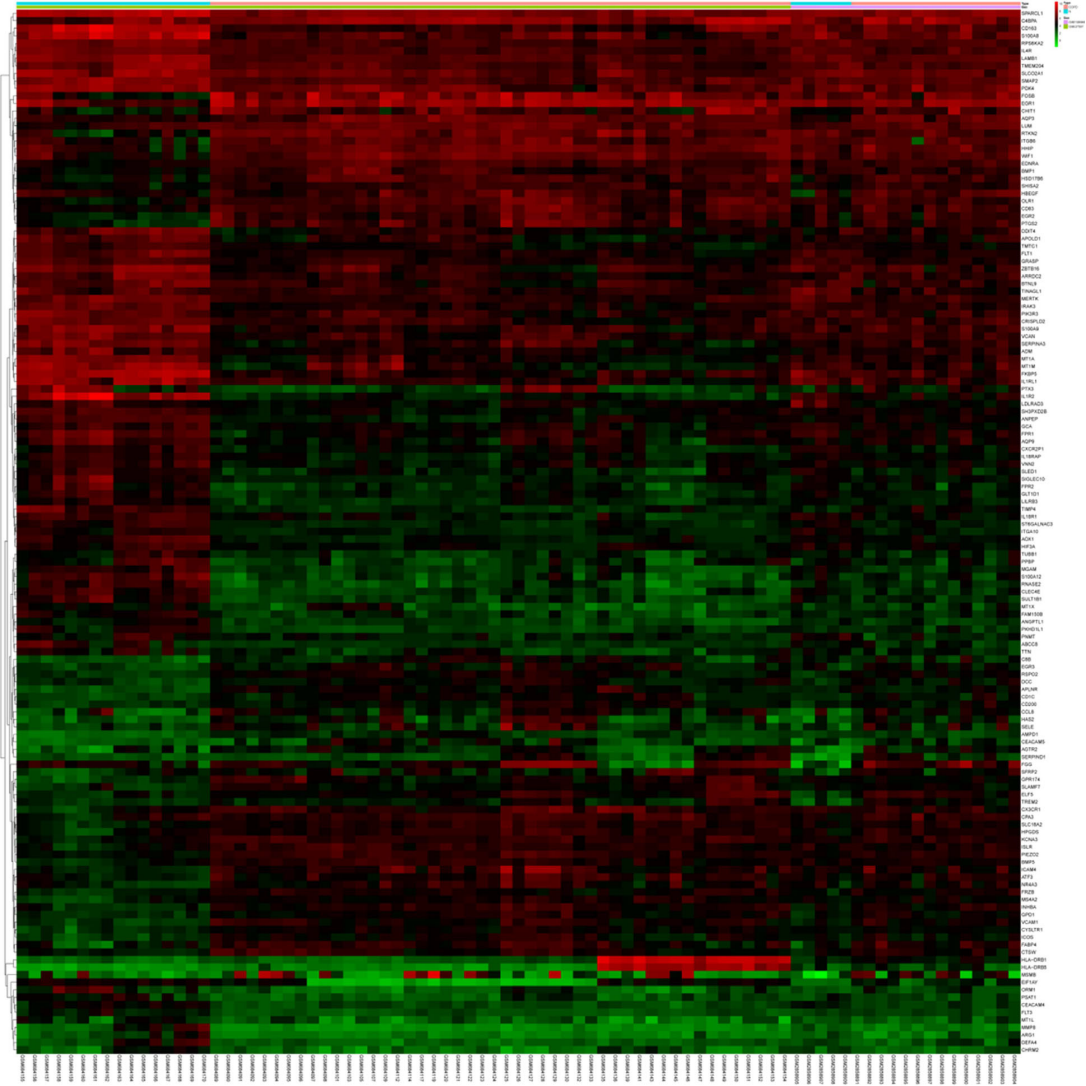
Hierarchical clustering heatmap of 139 DEGs screened on the basis of |FC| > 1 and a corrected *P* < 0.05. Red represents the up‐regulated DEGs, and green represents down‐regulated DEGs.

**Figure 3:**
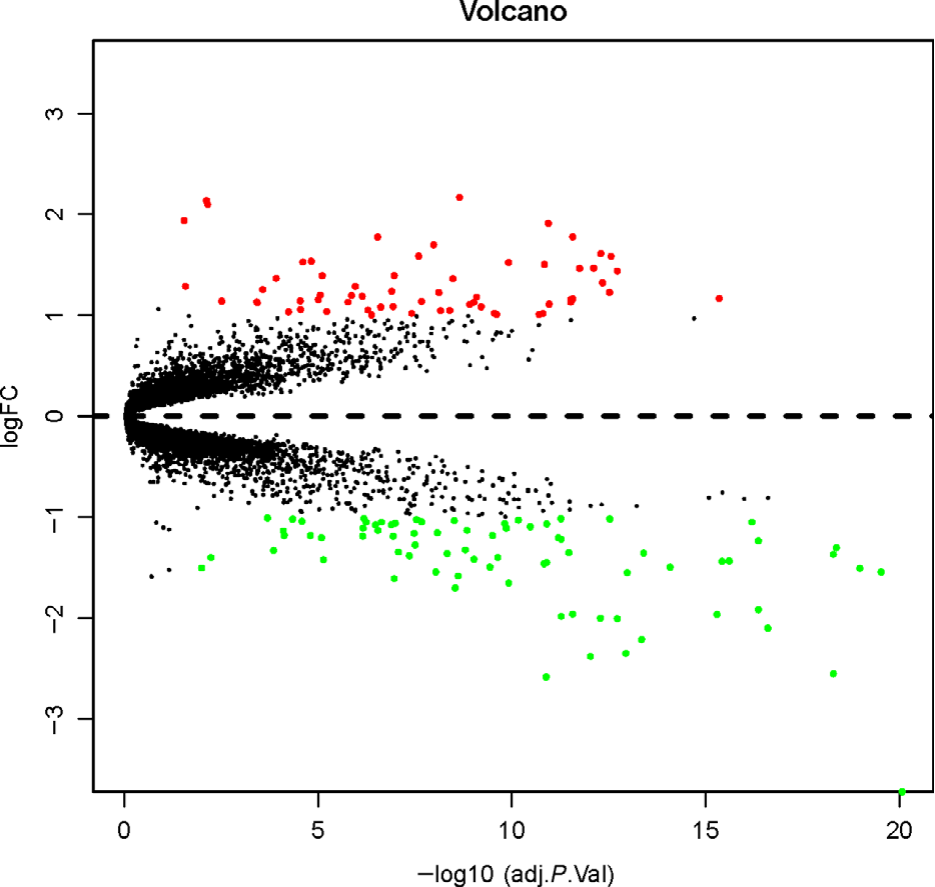
Volcano plots of differential gene expression data between two sets of samples. Red represents the up‐regulated DEGs, and green represents down‐regulated DEGs. adj.,*P*.Val, adjusted *P*‐value.

After batch normalization on the integrated dataset from GSE27597 and GSE106986 by the sva and limma packages, 139 DEGs were identified using the limma package (corrected *P* < 0.05; |log2FC| > 1) (Tables 1 and 2). The cluster heatmap of the top 139 DEGs is shown in Fig. 2. Among them, 62 genes were up‐regulated and 77 genes were down‐regulated, which is shown in Fig. 3.

**Table 1:** Screening up‐regulated and down‐regulated DEGs in COPD by integrated microarray

DEGs	Gene symbol
Up‐regulated (62 genes)	*PIEZO2*,* INHBA*,* VCAM1*,* RSPO2*,* CD1C*,* HSD17B6*,* AQP3*,* APLNR*,* GPD1*,* EGR2*,* KCNA*,* SLC18A2*,* FRZB*,* PTGS2*,* OLR1*,* EDNRA*,* SHISA2*,* ELF5*,* LUM*,* CYSLTR1*,* BMP5*,* HPGDS*,* MS4A2*,* DCC*,* FOSB*,* HHIP*,* MSMB*,* CD200*,* AMPD1*,* ICOS*,* RTKN2*,* CD83*,* FABP4*,* ISLR*,* GPR174*,* SLAMF7*,* WIF1*,* CTSW*,* CHIT1*,* CPA3*,* BMP1*,* EGR3*,* CX3CR1*,* EGR1*,* TREM2*,* ATF3*,* ICAM4*,* C8B*,* NR4A3*,* CCL8*,* HAS2*,* C4BPA*,* ITGB6*,* HBEGF*,* AGTR2*,* SERPIND1*,* CEACAM5*,* SFRP2*,* SELE*,* HLA‐DRB1*,* HLA‐DRB5*,* FGG*
Down‐regulated (77 genes)	*IL1R2*,* CEACAM4*,* ST6GALNAC3*,* ITGA10*,* MERTK*,* FKBP5*,* CLEC4E*,* LAMB1*,* MGAM*,* RPS6KA2*,* FLT1*,* IRAK3*,* SULT1B1*,* AOX1*,* SH3PXD2B*,* RNASE2*,* IL18R1*,* CD163*,* IL1RL1*,* GRASP*,* MT1A*,* S100A12*,* MMP8*,* GLT1D1*,* TMTC1*,* S100A8*,* IL4R*,* IL18RAP*,* FLT3*,* ANPEP*,* MT1M*,* SIGLEC10*,* SPARCL1*,* SMAP2*,* TIMP4*,* ANGPTL1*,* HIF3A*,* PKHD1L1*,* LILRB3*,* FPR1*,* SLED1*,* LDLRAD3*,* FAM150B*,* FPR2*,* ZBTB16*,* GCA*,* ARG1*,* CXCR2P1*,* S100A9*,* TMEM204*,* TINAGL1*,* ABCC8*,* VCAN*,* APOLD1*,* DDIT4*,* ARRDC2*,* SERPINA3*,* PIK3R3*,* ADM*,* PNMT*,* BTNL9*,* CRISPLD2*,* SLCO2A1*,* VNN2*,* TUBB1*,* PSAT1*,* PPBP*,* DEFA4*,* AQP9*,* TTN*,* PDK4*,* MT1L*,* MT1X*,* ORM1*,* CHRM2*,* PTX3*,* EIF1AY*

**Table 2:** Screening 139 DEGs in COPD by integrated microarray. AveExpr, average expression; FDR, false discovery rate

Gene symbol	logFC	AveExpr	*t*	*P*‐value	FDR
*IL1R2*	−3.724170635	4.910345092	−14.57070196	5.24E−25	8.38E−21
*CEACAM4*	−1.546190038	3.289938835	−14.11578934	3.65E−24	2.92E−20
*ST6GALNAC3*	−1.509348297	4.115922297	−13.72934365	1.93E−23	1.03E−19
*ITGA10*	−1.304452408	4.156071544	−13.3424854	1.05E−22	4.18E−19
*MERTK*	−1.371100619	5.743922084	−13.22669655	1.74E−22	4.99E−19
*FKBP5*	−2.553152066	5.964790869	−13.21010646	1.87E−22	4.99E−19
*CLEC4E*	−2.102592383	3.712865442	−12.2724444	1.22E−20	2.44E−17
*LAMB1*	−1.236900341	6.51036255	−12.11282186	2.51E−20	4.31E−17
*MGAM*	−1.918275604	3.743487329	−12.0971852	2.69E−20	4.31E−17
*RPS6KA2*	−1.051171425	6.235125074	−11.99287281	4.32E−20	6.28E−17
*FLT1*	−1.43735859	5.302047032	−11.6588263	1.98E−19	2.43E−16
*IRAK3*	−1.440362949	5.407265256	−11.53504535	3.48E−19	3.71E−16
*PIEZO2*	1.165120322	5.154801689	11.4846069	4.38E−19	4.38E−16
*SULT1B1*	−1.966128566	3.712292447	−11.44142282	5.34E−19	5.02E−16
*AOX1*	−1.498003567	4.380016656	−10.80239392	1.02E−17	8.14E−15
*SH3PXD2B*	−1.360072374	4.796109196	−10.45348254	5.15E−17	3.92E−14
*RNASE2*	−2.214352488	3.502846585	−10.41707921	6.11E−17	4.44E−14
*IL18R1*	−1.552709013	4.479557525	−10.21566115	1.56E−16	1.04E−13
*CD163*	−2.353331128	6.483508901	−10.18905044	1.77E−16	1.13E−13
*IL1RL1*	−2.008254697	5.747865421	−10.06701534	3.13E−16	1.88E−13
*INHBA*	1.437565757	4.698423572	10.06451042	3.17E−16	1.88E−13
*VCAM1*	1.58323448	4.54949487	9.975765691	4.80E−16	2.74E−13
*GRASP*	−1.019125221	5.351837423	−9.953904434	5.32E−16	2.93E−13
*RSPO2*	1.22508185	4.281279622	9.943524241	5.59E−16	2.98E−13
*CD1C*	1.320908918	4.048138575	9.847836259	8.75E−16	4.51E−13
*HSD17B6*	1.611303762	5.543589418	9.811225763	1.04E−15	5.03E−13
*MT1A*	−2.003809136	5.341700669	−9.802106469	1.08E−15	5.10E−13
*AQP3*	1.465444106	6.255672988	9.709215286	1.68E−15	7.66E−13
*S100A12*	−2.381911789	3.793860618	−9.658062064	2.13E−15	9.26E−13
*APLNR*	1.462761779	4.060794485	9.514044214	4.19E−15	1.76E−12
*GPD1*	1.162408188	4.425391991	9.418745073	6.56E−15	2.65E−12
*EGR2*	1.7756242	5.312493191	9.415498145	6.66E−15	2.65E−12
*MMP8*	−1.963376342	1.850422771	−9.410996945	6.80E−15	2.65E−12
*KCNA3*	1.127720579	5.181587794	9.376872801	7.99E−15	2.98E−12
*SLC18A2*	1.155076678	4.861252305	9.375465524	8.04E−15	2.98E−12
*GLT1D1*	−1.354253187	4.134707596	−9.333414043	9.80E−15	3.33E−12
*TMTC1*	−1.221922174	4.907718006	−9.2268097	1.62E−14	5.28E−12
*S100A8*	−1.984436486	6.732435285	−9.226677546	1.62E−14	5.28E−12
*IL4R*	−1.016737627	6.13438495	−9.220088063	1.67E−14	5.34E−12
*IL18RAP*	−1.20706302	4.403623117	−9.181902429	2.00E−14	6.26E−12
*FRZB*	1.108947075	4.570621342	9.054280215	3.64E−14	1.08E−11
*PTGS2*	1.909599543	5.385732252	9.037623232	3.94E−14	1.14E−11
*FLT3*	−1.068728329	2.955444457	−9.011875495	4.44E−14	1.24E−11
*OLR1*	1.516024757	5.630156756	9.006280553	4.56E−14	1.24E−11
*ANPEP*	−1.451754311	4.687966342	−9.001861447	4.66E−14	1.24E−11
*MT1M*	−2.585708871	5.454959847	−8.990902454	4.90E−14	1.29E−11
*SIGLEC10*	−1.464287775	4.29164058	−8.960779525	5.65E−14	1.46E−11
*EDNRA*	1.016233852	5.691095587	8.939250683	6.25E−14	1.56E−11
*SHISA2*	1.00660372	5.288052754	8.886393339	8.02E−14	1.94E−11
*SPARCL1*	−1.096814178	7.567105051	−8.760826399	1.45E−13	3.30E−11
*SMAP2*	−1.030733342	6.515681918	−8.593715399	3.17E−13	6.73E−11
*TIMP4*	−1.656076369	4.229601784	−8.456527093	6.04E−13	1.19E−10
*ELF5*	1.521617319	4.673219557	8.448782695	6.26E−13	1.21E−10
*ANGPTL1*	−1.111036176	3.340161894	−8.413321275	7.39E−13	1.37E−10
*HIF3A*	−1.065614158	4.526630285	−8.386705336	8.38E−13	1.52E−10
*PKHD1L1*	−1.402091953	3.533210448	−8.281317436	1.37E−12	2.29E−10
*LUM*	1.008125744	6.388824425	8.261829056	1.50E−12	2.45E−10
*CYSLTR1*	1.017621995	4.334713339	8.231060286	1.74E−12	2.78E−10
*LILRB3*	−1.182035723	3.930324211	−8.201110624	2.00E−12	3.11E−10
*FPR1*	−1.497358339	5.123265733	−8.165184027	2.36E−12	3.63E−10
*BMP5*	1.082572	4.856866963	8.039056504	4.26E−12	6.14E−10
*HPGDS*	1.178976758	4.9661482	7.971057179	5.85E−12	8.07E−10
*SLED1*	−1.418643068	4.176703046	−7.93529438	6.92E−12	9.42E−10
*MS4A2*	1.127545704	4.501069641	7.934255426	6.95E−12	9.42E−10
*DCC*	1.107054924	4.066396917	7.874112823	9.20E−12	1.21E−9
*LDLRAD3*	−1.132183539	4.777010972	−7.827670594	1.14E−11	1.42E−9
*FAM150B*	−1.326763929	3.661484534	−7.801467295	1.29E−11	1.57E−9
*FOSB*	2.16754811	6.605909434	7.722677313	1.86E−11	2.20E−9
*FPR2*	−1.585779616	4.015151074	−7.702560188	2.04E−11	2.40E−9
*ZBTB16*	−1.704030574	5.808698383	−7.660799671	2.48E−11	2.87E−9
*GCA*	−1.036962474	4.831056628	−7.646120016	2.65E−11	3.03E−9
*HHIP*	1.361681455	6.338309941	7.624501498	2.93E−11	3.28E−9
*CD200*	1.045448111	4.105658515	7.579848564	3.61E−11	3.95E−9
*ARG1*	−1.363394352	2.024018729	−7.54254425	4.29E−11	4.60E−9
*AMPD1*	1.043721979	3.103940463	7.443735113	6.77E−11	6.85E−9
*ICOS*	1.222541378	4.277643563	7.41874473	7.59E−11	7.59E−9
*CXCR2P1*	−1.15616615	4.439580145	−7.399724607	8.29E−11	8.23E−9
*S100A9*	−1.545635339	5.747693368	−7.378920244	9.12E−11	9.01E−9
*RTKN2*	1.696929199	6.166705232	7.344341435	1.07E−10	1.03E−8
*TMEM204*	−1.048529786	6.26406167	−7.165974961	2.43E−10	2.11E−8
*CD83*	1.134658456	5.71435664	7.163495775	2.45E−10	2.12E−8
*FABP4*	1.58545563	4.778254935	7.12319994	2.95E−10	2.51E−8
*TINAGL1*	−1.026326535	5.532226715	−7.093847539	3.38E−10	2.84E−8
*ABCC8*	−1.278473261	3.740007718	−7.069781506	3.77E−10	3.09E−8
*VCAN*	−1.162194417	5.676393323	−7.050563618	4.11E−10	3.29E−8
*ISLR*	1.017944512	5.018753667	7.010420181	4.94E−10	3.80E−8
*APOLD1*	−1.385170588	5.098238511	−6.976021904	5.78E−10	4.35E−8
*DDIT4*	−1.34772118	5.257280573	−6.808572358	1.23E−9	8.33E−8
*ARRDC2*	−1.061269085	5.499220509	−6.762488227	1.52E−9	1.01E−7
*GPR174*	1.391314128	4.486280437	6.747714106	1.63E−9	1.07E−7
*SERPINA3*	−1.612709918	5.391772667	−6.745585747	1.64E−9	1.07E−7
*PIK3R3*	−1.190376757	5.660292101	−6.728449631	1.77E−9	1.14E−7
*SLAMF7*	1.083604552	4.392800911	6.727219831	1.78E−9	1.14E−7
*WIF1*	1.237156067	6.258872842	6.708747667	1.94E−9	1.24E−7
*ADM*	−1.075662424	5.196278444	−6.701236397	2.00E−9	1.28E−7
*PNMT*	−1.051631588	4.068574241	−6.553848929	3.88E−9	2.30E−7
*CTSW*	1.07957302	4.747480058	6.545368949	4.04E−9	2.38E−7
*BTNL9*	−1.132836349	5.578696855	−6.494296937	5.07E−9	2.82E−7
*CHIT1*	1.774209888	5.594573377	6.490067624	5.17E−9	2.87E−7
*CRISPLD2*	−1.078448778	5.7156417	−6.459148182	5.93E−9	3.26E−7
*CPA3*	1.002710147	5.278769406	6.398904433	7.75E−9	4.12E−7
*BMP1*	1.049814522	5.450758141	6.3426456	9.95E−9	5.12E−7
*SLCO2A1*	−1.051736858	6.743524273	−6.312993603	1.13E−8	5.69E−7
*VNN2*	−1.015536891	4.57616008	−6.274690529	1.34E−8	6.55E−7
*TUBB1*	−1.110536854	3.804924924	−6.264199999	1.41E−8	6.82E−7
*PSAT1*	−1.190066031	3.005323949	−6.258737184	1.44E−8	6.94E−7
*EGR3*	1.186935902	4.097335491	6.248573488	1.51E−8	7.22E−7
*CX3CR1*	1.284828947	5.212105592	6.144966898	2.38E−8	1.08E−6
*EGR1*	1.194905541	7.538328018	6.08564001	3.08E−8	1.35E−6
*TREM2*	1.128703086	3.987081843	6.03163178	3.90E−8	1.67E−6
*ATF3*	1.034162475	4.884273698	5.696410877	1.65E−7	5.92E−6
*PPBP*	−1.424569764	3.860588827	−5.646955582	2.04E−7	7.16E−6
*ICAM4*	1.390911425	5.019641081	5.625030952	2.24E−7	7.74E−6
*DEFA4*	−1.206319405	2.718323142	−5.614366702	2.34E−7	8.06E−6
*C8B*	1.199289417	3.724442441	5.595043977	2.54E−7	8.65E−6
*NR4A3*	1.153028606	4.646253713	5.56155394	2.93E−7	9.79E−6
*CCL8*	1.533954481	3.932933151	5.448623389	4.71E−7	1.48E−5
*AQP9*	−1.182290805	4.835881555	−5.430616006	5.08E−7	1.56E−5
*HAS2*	1.525864945	3.562127425	5.306311934	8.51E−7	2.44E−5
*TTN*	−1.043207507	3.817413024	−5.293238479	8.98E−7	2.56E−5
*C4BPA*	1.055176798	7.356691544	5.268476209	9.95E−7	2.79E−5
*ITGB6*	1.140444449	6.076736655	5.260091655	1.03E−6	2.86E−5
*PDK4*	−1.021408815	6.733080202	−5.134267178	1.72E−6	4.47E−5
*HBEGF*	1.032852155	5.680806271	5.061817586	2.31E−6	5.73E−5
*MT1L*	−1.182918274	2.526143932	−4.990697548	3.08E−6	7.38E−5
*MT1X*	−1.137570423	3.368378253	−4.974584006	3.29E−6	7.77E−5
*AGTR2*	1.364930071	3.524442993	4.842112874	5.57E−6	0.000119643
*ORM1*	−1.331793001	2.918107873	−4.800748967	6.56E−6	0.000138298
*CHRM2*	−1.011281909	2.379732249	−4.689445874	1.01E−5	0.000200001
*SERPIND1*	1.253485683	3.227485742	4.600275561	1.43E−5	0.000265554
*CEACAM5*	1.121861431	3.16229753	4.495719542	2.14E−5	0.000368719
*SFRP2*	1.129656517	4.14961175	4.486856217	2.21E−5	0.000377179
*SELE*	1.138034748	3.853029685	3.80214458	0.00026654	0.003035211
*PTX3*	−1.402248844	4.135304651	−3.574413207	0.000577009	0.005747801
*HLA‐DRB1*	2.096264176	4.01015487	3.496867141	0.000745469	0.007027456
*HLA‐DRB5*	2.134896841	3.007061623	3.473802838	0.000803932	0.007481531
*EIF1AY*	−1.506138016	3.020677733	−3.35747922	0.00117078	0.010029593
*FGG*	1.286056757	4.918716551	2.965432506	0.003905804	0.026040868
*MSMB*	1.936339164	3.146192242	2.931036227	0.00432076	0.028265267

### GO enrichment analysis of DEGs

**Figure 4:**
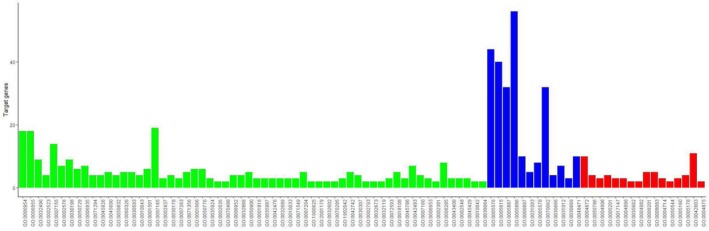
GO enrichment analysis of DEGs in COPD. GO analysis divided DEGs into three functional groups: BPs, cell composition and MF. Green represents BP category, blue represents cell composition category and red represents MF category.

**Figure 5:**
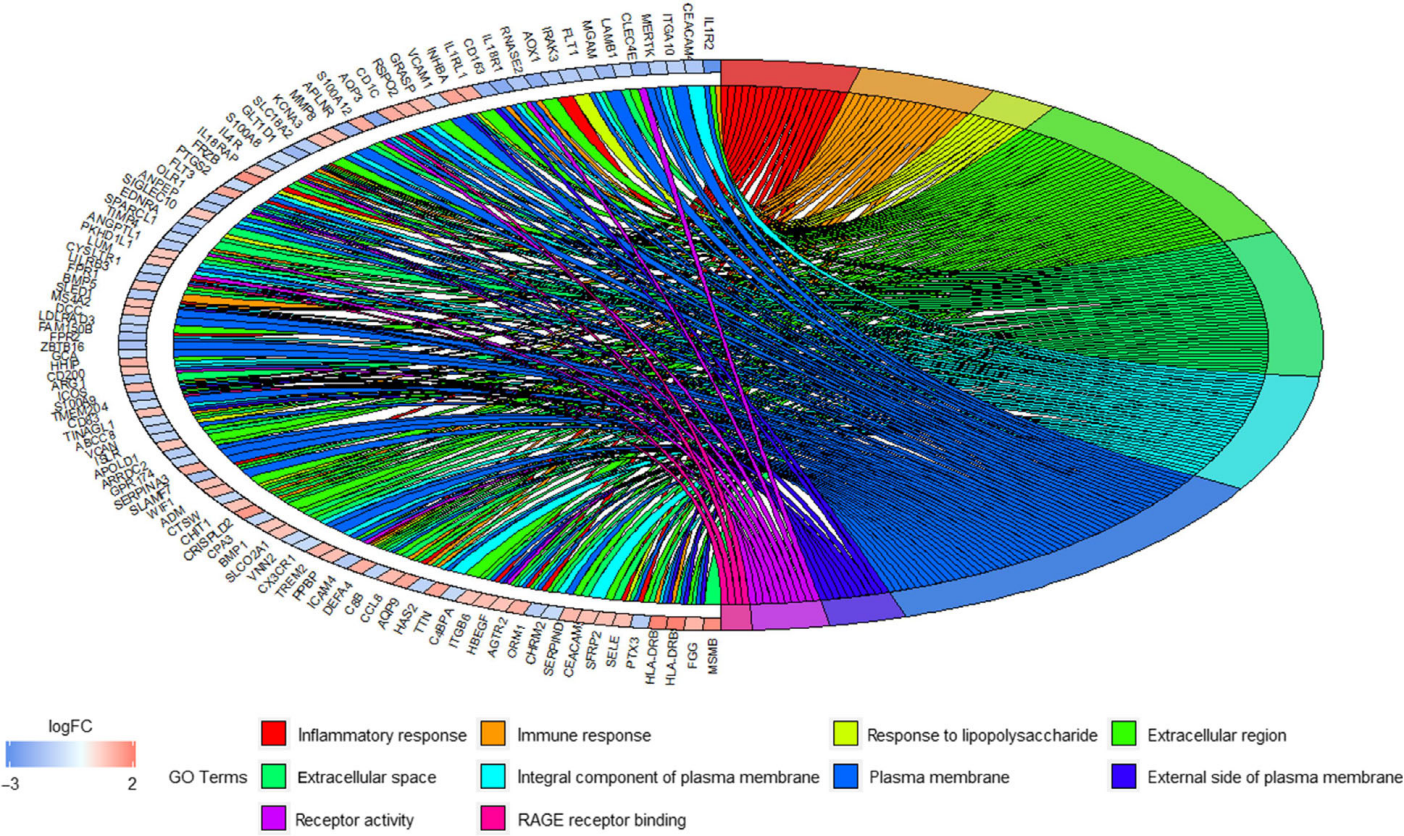
Distribution of DEGs in COPD for the most significant GO‐enriched functions.

GO analysis was done on the DEGs against BP, MF and CC terms. Biological annotation of the DEGs with COPD was identified using the david online analysis tool. As shown in Fig. 4, GO functional enrichments of the DEGs with a *P*‐value <0.05 were obtained. Significant results of the GO enrichment analysis of the DEGs associated with COPD were shown in Table 3. In the BP category, the DEGs were mainly involved in inflammatory response, immune response and response to lipopolysaccharide. In the MF category, the DEGs were mainly enriched in receptor activity and receptor for advanced glycation end products (RAGE) receptor binding. In the CC category, the DEGs were mainly involved in the extracellular region and space, the integral component of the plasma membrane, the plasma membrane and the external side of the plasma membrane (Fig. 5).

**Table 3:** GO analysis of DEGs associated with COPD

Term ID	Category	Description	Count	*P*‐value	Bonferroni
GO:0006954	BP	Inflammatory response	18	9.11E−9	0.0000088
GO:0006955	BP	Immune response	18	4.26E−8	0.0000412
GO:0032496	BP	Response to lipopolysaccharide	9	0.0000513	0.04832
GO:0002523	BP	Leukocyte migration involved in inflammatory response	4	0.0000782	0.072795
GO:0007155	BP	Cell adhesion	14	0.0000787	0.073195
GO:0002576	BP	Platelet degranulation	7	0.000175	0.155654
GO:0030198	BP	Extracellular matrix organization	9	0.000178	0.157584
GO:0050729	BP	Positive regulation of inflammatory response	6	0.000292	0.245789
GO:0006935	BP	Chemotaxis	7	0.000438	0.344834
GO:0071294	BP	Cellular response to zinc ion	4	0.000438	0.345355
GO:0045926	BP	Negative regulation of growth	4	0.000438	0.345355
GO:0045600	BP	Positive regulation of fat cell differentiation	5	0.00053	0.400817
GO:0050832	BP	Defense response to fungus	4	0.001263	0.705093
GO:0060326	BP	Cell chemotaxis	5	0.001801	0.824787
GO:0030593	BP	Neutrophil chemotaxis	5	0.001906	0.841641
GO:0010043	BP	Response to zinc ion	4	0.002927	0.941058
GO:0001501	BP	Skeletal system development	6	0.004843	0.990808
GO:0007165	BP	Signal transduction	19	0.00503	0.992337
GO:0002437	BP	Inflammatory response to antigenic stimulus	3	0.0062	0.997541
GO:0030178	BP	Negative regulation of Wnt signaling pathway	4	0.008258	0.999668
GO:0007263	BP	Nitric oxide‐mediated signal transduction	3	0.009889	0.999932
GO:0071356	BP	Cellular response to tumor necrosis factor	5	0.011676	0.999988
GO:0001666	BP	Response to hypoxia	6	0.012312	0.999994
GO:0050776	BP	Regulation of immune response	6	0.014105	0.999999
GO:0035924	BP	Cellular response to vascular endothelial growth factor stimulus	3	0.014331	0.999999
GO:0002035	BP	Brain renin‐angiotensin system	2	0.015897	1
GO:0070488	BP	Neutrophil aggregation	2	0.015897	1
GO:0006952	BP	Defense response	4	0.016421	1
GO:0032868	BP	Response to insulin	4	0.016421	1
GO:0050900	BP	Leukocyte migration	5	0.016519	1
GO:0001816	BP	Cytokine production	3	0.016818	1
GO:0035987	BP	Endodermal cell differentiation	3	0.019474	1
GO:0042476	BP	Odontogenesis	3	0.019474	1
GO:0032689	BP	Negative regulation of interferon‐gamma production	3	0.020864	1
GO:0010033	BP	Response to organic substance	3	0.020864	1
GO:0071549	BP	Cellular response to dexamethasone stimulus	3	0.022294	1
GO:0007204	BP	Positive regulation of cytosolic calcium ion concentration	5	0.022427	1
GO:1900625	BP	Positive regulation of monocyte aggregation	2	0.023751	1
GO:2001179	BP	Regulation of interleukin‐10 secretion	2	0.023751	1
GO:0032602	BP	Chemokine production	2	0.023751	1
GO:0070295	BP	Renal water absorption	2	0.023751	1
GO:1902042	BP	Negative regulation of extrinsic apoptotic signaling pathway via death domain receptors	3	0.0284	1
GO:0042742	BP	Defense response to bacterium	5	0.028971	1
GO:0030307	BP	Positive regulation of cell growth	4	0.029638	1
GO:0002793	BP	Positive regulation of peptide secretion	2	0.031543	1
GO:0032673	BP	Regulation of interleukin‐4 production	2	0.031543	1
GO:0032119	BP	Sequestering of zinc ion	2	0.031543	1
GO:0072593	BP	Reactive oxygen species metabolic process	3	0.031676	1
GO:0018108	BP	Peptidyl‐tyrosine phosphorylation	5	0.034254	1
GO:0045786	BP	Negative regulation of cell cycle	3	0.035093	1
GO:0042493	BP	Response to drug	7	0.03518	1
GO:0007160	BP	Cell‐matrix adhesion	4	0.035316	1
GO:0006953	BP	Acute‐phase response	3	0.038646	1
GO:0002381	BP	Immunoglobulin production involved in immunoglobulin‐mediated immune response	2	0.039273	1
GO:0008285	BP	Negative regulation of cell proliferation	8	0.039604	1
GO:0043408	BP	Regulation of mitogen‐activated protein kinase cascade	3	0.042329	1
GO:0002548	BP	Monocyte chemotaxis	3	0.044219	1
GO:0045429	BP	Positive regulation of nitric oxide biosynthetic process	3	0.046139	1
GO:0010042	BP	Response to manganese ion	2	0.046942	1
GO:0038084	BP	Vascular endothelial growth factor signaling pathway	2	0.046942	1
GO:0005576	CC	Extracellular region	44	2.35E−14	3.22E−12
GO:0005615	CC	Extracellular space	40	4.29E−14	5.89E−12
GO:0005887	CC	Integral component of plasma membrane	32	2.71E−8	0.00000371
GO:0005886	CC	Plasma membrane	56	0.00000132	0.000181
GO:0009897	CC	External side of plasma membrane	10	0.0000284	0.003882
GO:0031093	CC	Platelet alpha‐granule lumen	5	0.000713	0.093083
GO:0005578	CC	Proteinaceous extracellular matrix	8	0.003704	0.398509
GO:0070062	CC	Extracellular exosome	32	0.011945	0.807247
GO:0030666	CC	Endocytic vesicle membrane	4	0.012698	0.826358
GO:0031012	CC	Extracellular matrix	7	0.02228	0.954358
GO:0030669	CC	Clathrin‐coated endocytic vesicle membrane	3	0.03651	0.993875
GO:0048471	CC	Perinuclear region of cytoplasm	10	0.039931	0.996238
GO:0004872	MF	Receptor activity	10	0.0000321	0.009231
GO:0050786	MF	RAGE receptor binding	4	0.0000611	0.017515
GO:0004908	MF	Interleukin‐1 receptor activity	3	0.001097	0.271869
GO:0005201	MF	Extracellular matrix structural constituent	4	0.013171	0.97833
GO:0017147	MF	Wnt protein binding	3	0.02166	0.998215
GO:0004896	MF	Cytokine receptor activity	3	0.028658	0.999776
GO:0035662	MF	Toll‐like receptor 4 binding	2	0.029063	0.999801
GO:0004982	MF	*N*‐formyl peptide receptor activity	2	0.029063	0.999801
GO:0008201	MF	Heparin binding	5	0.030385	0.999866
GO:0008083	MF	Growth factor activity	5	0.031597	0.999907
GO:0004714	MF	Transmembrane receptor protein tyrosine kinase activity	3	0.031677	0.999909
GO:0050544	MF	Arachidonic acid binding	2	0.036196	0.999976
GO:0005160	MF	Transforming growth factor‐beta receptor binding	3	0.03807	0.999987
GO:0005178	MF	Integrin binding	4	0.042233	0.999996
GO:0042803	MF	Protein homodimerization activity	11	0.042565	0.999997
GO:0004875	MF	Complement receptor activity	2	0.043278	0.999997

### KEGG pathway analysis of DEGs

**Figure 6:**
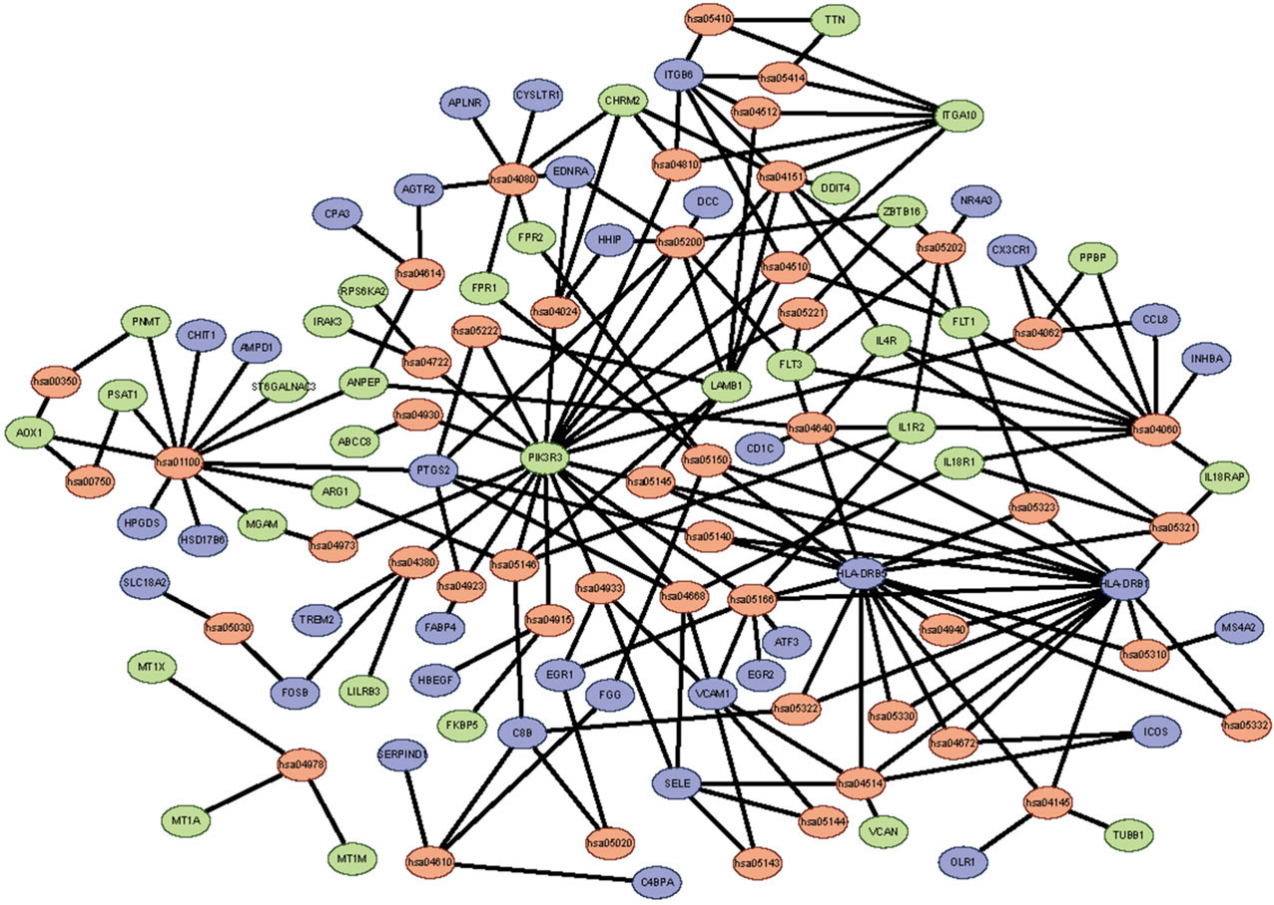
The significant KEGG pathways enrichment of DEGs. Green represents down‐regulated DEGs, blue represents up‐regulated DEGs and red represents the signaling pathway.

We analyzed the DEGs using the KOBAS 3.0 online analysis database. As shown in Table 4, the DEGs were enriched in 48 pathways, especially hematopoietic cell lineage and cytokine‐cytokine receptor interaction. The genes and pathway nodes are represented by cytoscape version 3.6.1 software that was used to calculate the topological characteristics of the network and determine each node (Fig. 6).

**Table 4:** KEGG pathway analysis of DEGs associated with COPD

ID	Pathway	Gene count	Corrected *P*‐value	DEGs
hsa04640	Hematopoietic cell lineage	7	4.07E−6	*FLT3*,* IL4R*,* HLA‐DRB1*,* HLA‐DRB5*,* IL1R2*,* CD1C*,* ANPEP*
hsa04060	Cytokine‐cytokine receptor interaction	10	4.07E−6	*FLT3*,* FLT1*,* CCL8*,* IL4R*,* INHBA*,* CX3CR1*,* IL1R2*,* IL18RAP*,* PPBP*,* IL18R1*
hsa05150	*Staphylococcus aureus* infection	5	0.000147	*HLA‐DRB1*,* FPR2*,* FGG*,* FPR1*,* HLA‐DRB5*
hsa05166	Human T‐lymphotropic virus type 1 infection	8	0.000174	*PIK3R3*,* HLA‐DRB1*,* ATF3*,* HLA‐DRB5*,* VCAM1*,* IL1R2*,* EGR2*,* EGR1*
hsa05321	Inflammatory bowel disease	5	0.000174	*HLA‐DRB1*,* IL18RAP*,* IL4R*,* HLA‐DRB5*,* IL18R1*
hsa04514	Cell adhesion molecules	6	0.000456	*SELE*,* HLA‐DRB1*,* ICOS*,* HLA‐DRB5*,* VCAM1*,* VCAN*
hsa04151	Phosphoinositide 3‐kinase‐Akt signaling pathway	8	0.000732	*CHRM2*,* PIK3R3*,* ITGA10*,* IL4R*,* LAMB1*,* DDIT4*,* ITGB6*,* FLT1*
hsa05146	Amoebiasis	5	0.000732	*C8B*,* PIK3R3*,* ARG1*,* LAMB1*,* IL1R2*
hsa04668	Tumor necrosis factor signaling pathway	5	0.001006	*VCAM1*,* PIK3R3*,* SELE*,* PTGS2*,* IL18R1*
hsa04080	Neuroactive ligand‐receptor interaction	7	0.00112	*CHRM2*,* FPR2*,* AGTR2*,* APLNR*,* EDNRA*,* CYSLTR1*,* FPR1*
hsa05200	Pathways in cancer	8	0.001401	*DCC*,* FLT3*,* ZBTB16*,* LAMB1*,* PIK3R3*,* HHIP*,* PTGS2*,* EDNRA*
hsa04614	Renin‐angiotensin system	3	0.00144	*CPA3*,* AGTR2*,* ANPEP*
hsa04610	Complement and coagulation cascades	4	0.002727	*FGG*,* C8B*,* C4BPA*,* SERPIND1*
hsa05310	Asthma	3	0.003037	*HLA‐DRB1*,* HLA‐DRB5*,* MS4A2*
hsa00750	Vitamin B_6_ metabolism	2	0.003805	*PSAT1*,* AOX1*
hsa05202	Transcriptional misregulation in cancer	5	0.005115	*FLT3*,* ZBTB16*,* FLT1*,* NR4A3*,* IL1R2*
hsa04933	AGE‐RAGE signaling pathway in diabetic complications	4	0.005115	*VCAM1*,* PIK3R3*,* SELE*,* EGR1*
hsa04510	Focal adhesion	5	0.007653	*PIK3R3*,* ITGA10*,* ITGB6*,* LAMB1*,* FLT1*
hsa04672	Intestinal immune network for IgA production	3	0.007653	*HLA‐DRB1*,* ICOS*,* HLA‐DRB5*
hsa05145	Toxoplasmosis	4	0.007715	*HLA‐DRB1*,* PIK3R3*,* LAMB1*,* HLA‐DRB5*
hsa04978	Mineral absorption	3	0.007715	*MT1X*,* MT1A*,* MT1M*
hsa04923	Regulation of lipolysis in adipocytes	3	0.009035	*PIK3R3*,* PTGS2*,* FABP4*
hsa05221	Acute myeloid leukemia	3	0.009074	*FLT3*,* ZBTB16*,* PIK3R3*
hsa04380	Osteoclast differentiation	4	0.009515	*LILRB3*,* PIK3R3*,* FOSB*,* TREM2*
hsa01100	Metabolic pathways	12	0.00997	*PSAT1*,* HPGDS*,* ARG1*,* ST6GALNAC3*,* AOX1*,* PNMT*,* HSD17B6*,* AMPD1*,* PTGS2*,* CHIT1*,* MGAM*,* ANPEP*
hsa04145	Phagosome	4	0.015537	*HLA‐DRB1*,* TUBB1*,* HLA‐DRB5*,* OLR1*
hsa05140	Leishmaniasis	3	0.015831	*HLA‐DRB1*,* PTGS2*,* HLA‐DRB5*
hsa04512	Extracellular matrix‐receptor interaction	3	0.02016	*ITGA10*,* ITGB6*,* LAMB1*
hsa05410	Hypertrophic cardiomyopathy	3	0.02016	*TTN*,* ITGA10*,* ITGB6*
hsa05222	Small‐cell lung cancer	3	0.021463	*PIK3R3*,* PTGS2*,* LAMB1*
hsa05414	Dilated cardiomyopathy	3	0.023455	*TTN*,* ITGA10*,* ITGB6*
hsa05323	Rheumatoid arthritis	3	0.023455	*HLA‐DRB1*,* FLT1*,* HLA‐DRB5*
hsa04062	Chemokine signaling pathway	4	0.023572	*CX3CR1*,* PIK3R3*,* CCL8*,* PPBP*
hsa04915	Estrogen signaling pathway	3	0.027527	*FKBP5*,* PIK3R3*,* HBEGF*
hsa04024	cAMP signaling pathway	4	0.027527	*CHRM2*,* PIK3R3*,* HHIP*,* EDNRA*
hsa04810	Regulation of actin cytoskeleton	4	0.032602	*CHRM2*,* PIK3R3*,* ITGA10*,* ITGB6*
hsa00350	Tyrosine metabolism	2	0.032602	*AOX1*,* PNMT*
hsa05020	Prion diseases	2	0.032602	*C8B*,* EGR1*
hsa05143	African trypanosomiasis	2	0.032602	*VCAM1*,* SELE*
hsa05330	Allograft rejection	2	0.038438	*HLA‐DRB1*,* HLA‐DRB5*
hsa04722	Neurotrophin signaling pathway	3	0.038438	*IRAK3*,* PIK3R3*,* RPS6KA2*
hsa05332	Graft‐versus‐host disease	2	0.042328	*HLA‐DRB1*,* HLA‐DRB5*
hsa04940	Type I diabetes mellitus	2	0.04503	*HLA‐DRB1*,* HLA‐DRB5*
hsa04973	Carbohydrate digestion and absorption	2	0.047746	*MGAM*,* PIK3R3*
hsa05322	Systemic lupus erythematosus	3	0.048708	*HLA‐DRB1*,* C8B*,* HLA‐DRB5*
hsa04930	Type II diabetes mellitus	2	0.049139	*ABCC8*,* PIK3R3*
hsa05144	Malaria	2	0.049139	*VCAM1*,* SELE*
hsa05030	Cocaine addiction	2	0.049139	*SLC18A2*,* FOSB*

### PPI network analysis of DEGs

**Figure 7:**
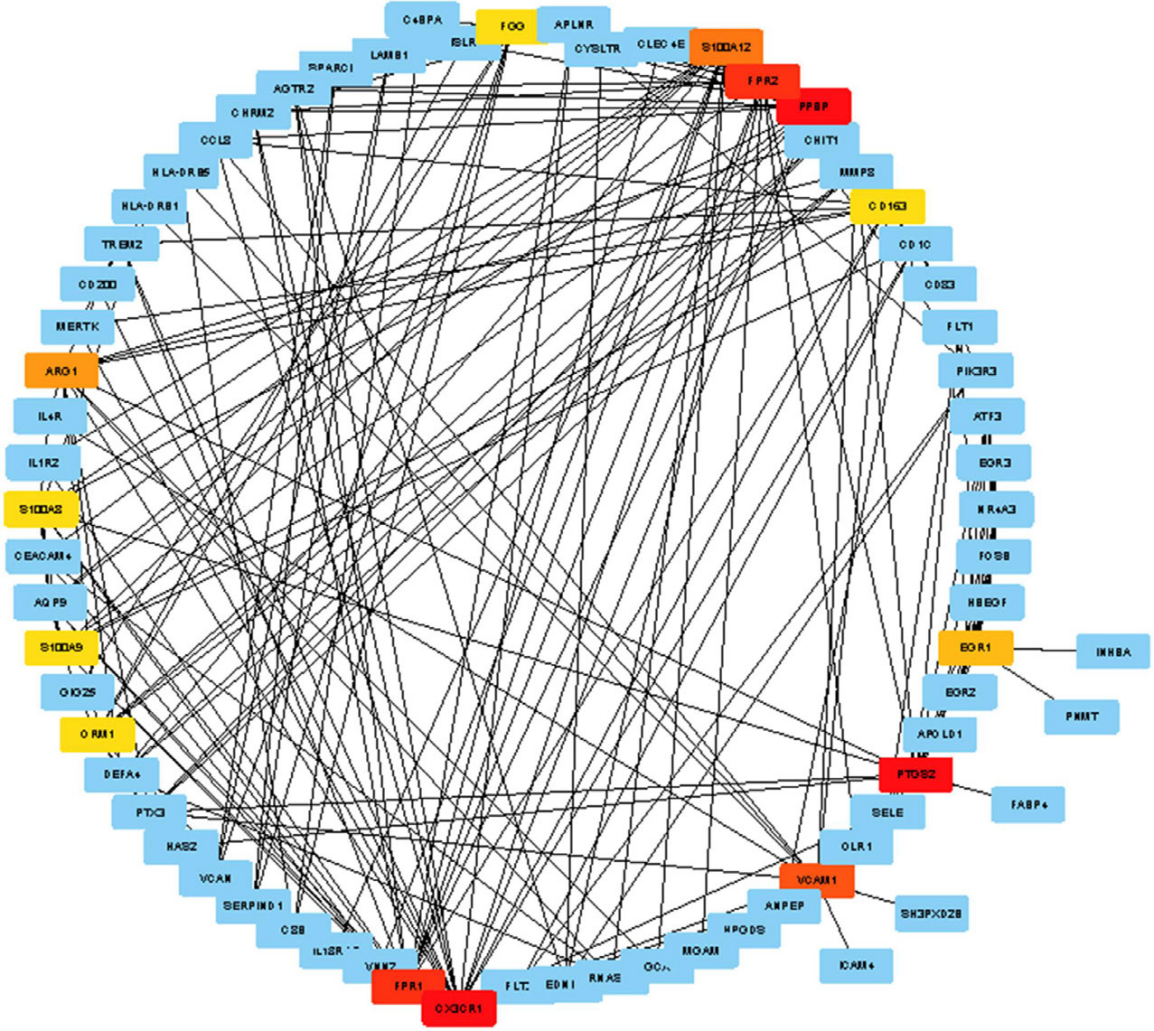
PPI network and Hub DEGs. Hub DEGs were identified with cytoscape version 3.6.1 with cytoHubba plugin, according to the rank of connection degree (number) for each gene, which is represented by the different degrees of color (from red to yellow): the role of the gene is greater in the PPI network with the darker color of the gene. Red, saffron yellow and yellow represent Hub DEGs.

**Figure 8:**
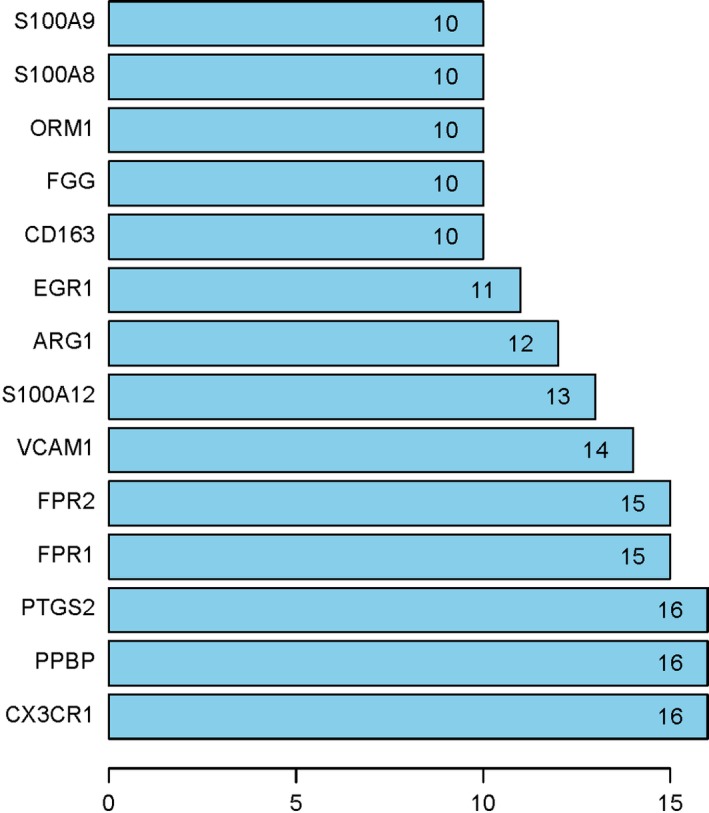
PPI network identified Hub DEGs. Numbers represent connection points of the 14 Hub genes identified by the cytoHubba plugin.

The 139 DEGs were applied for construction of PPI networks using STRING. After removing the discrete and partially connected nodes, the PPI network data of DEGs were imported into the cytoHubba of cytoscape version 3.6.1, and a complex network of the DEGs was constructed. As shown in Figs 7 and 8, 14 Hub DEGs were obtained, including *C‐X3‐C motif chemokine receptor 1* (*CX3CR1*), *proplatelet basic protein* (*PPBP*), *prostaglandin‐endoperoxide synthase 2* (*PTGS2*), *formyl peptide receptor 1* (*FPR1*), *formyl peptide receptor 2* (*FPR2*), *vascular cell adhesion molecule 1* (*VCAM1*), *S100 calcium binding protein A12* (*S100A12*), *arginase 1* (*ARG1*), *early growth response 1* (*EGR1*), *CD163*,* fibrinogen gamma chain* (*FGG*), *orosomucoid 1* (*ORM1*), *S100 calcium binding protein A8* (*S100A8*) and *S100 calcium binding protein A9* (*S100A9*).

### wgcna network construction in lung tissues

**Figure 9:**
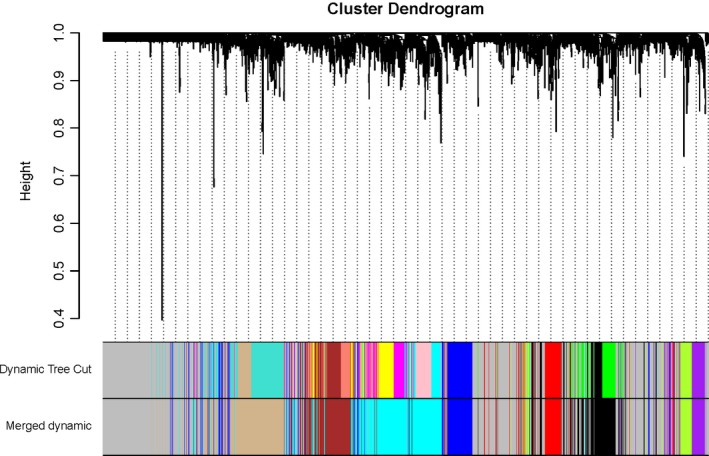
Network construction identifies distinct modules of coexpressed genes. The network was constructed using the lung tissue expression dataset of GSE27597 and GSE106986. The cluster dendrogram was produced by average linkage hierarchical clustering of genes using 1 − topological overlap as dissimilarity measure. Modules (Dynamic Tree Cut) and similarly merged modules (Merged dynamic) of coexpressed genes were assigned colors corresponding to the branches indicated by the horizontal bar beneath the dendrogram (merged cut height = 0.25).

A wgcna network was first constructed using lung tissue expression data from cohorts GSE27597 and GSE106986, independent of COPD status and smoking status (ever/current smoking versus nonsmoking). A total of 2942 DEGs were selected (a corrected *P* < 0.05) and subsequently used to identify modules of coexpressed genes using a hierarchical clustering procedure. The corresponding branches of the resulting dynamic clustering tree and module are shown as colored bands underneath the cluster tree. We then merged the highly similar dynamic clustering modules into the merged dynamic modules (cut height = 0.25) (Fig. 9). We identified nine modules ranging in size from 113 genes in the Purple module to 1081 in the Grey module. A module eigengene, a weighted average of the module gene expression profiles, was used to summarize the expression profiles of transcripts in a given module through their first principal component.

### Coexpression modules associated with COPD

**Figure 10:**
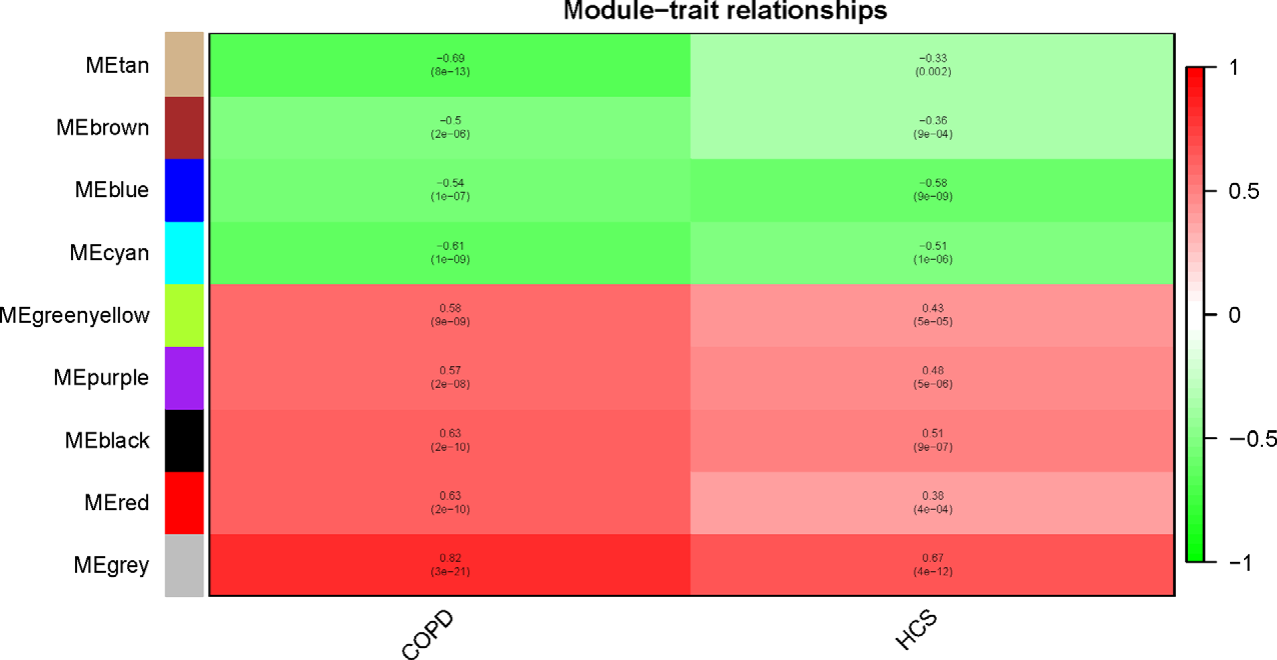
wgcna heatmap. Using the default parameter setting and all DEGs (*n* = 2942), we identified nine gene modules using wgcna that were positively or negatively associated with COPD and smoking trait. Each row corresponds to a module eigengene and each column to a clinical trait (COPD and smoking status). Positive associations are red, and negative associations are green. HCS, history of smoking.

To pinpoint modules associated with COPD and smoking status, we analyzed the association of each of the nine module eigengenes with the two traits. As shown in Fig. 10 and Table 5, all nine modules were significantly correlated with COPD and smoking status. Four modules were negatively associated with COPD and smoking status, marked Tan, Brown, Blue and Cyan, indicating that genes in these modules were predominantly down‐regulated in COPD cases and those who had a history of smoking. However, five modules, in Green yellow, Purple, Black, Red and Grey, were positively associated with COPD cases and smoking status, showing that genes in these modules are predominantly up‐regulated with the traits.

**Table 5:** Correlation of module eigengene with COPD and smoking status traits

wgcna modules	Gene number	Merged COPD dataset		Smoking status	
		Correlation	*P*‐value	Correlation	*P*‐value
Tan	353	−0.69	8E−13	−0.33	0.002
Brown	244	−0.5	2E−6	−0.36	9E−4
Blue	202	−0.54	1E−7	−0.58	9E−9
Cyan	467	−0.61	1E−9	−0.51	1E−6
Green yellow	105	0.58	9E−9	0.43	5E−5
Purple	113	0.57	2E−8	0.48	5E−6
Black	256	0.63	2E−10	0.51	9E−7
Red	121	0.63	2E−10	0.38	4E−4
Grey	1081	0.82	3E−21	0.67	4E−12

**Figure 11:**
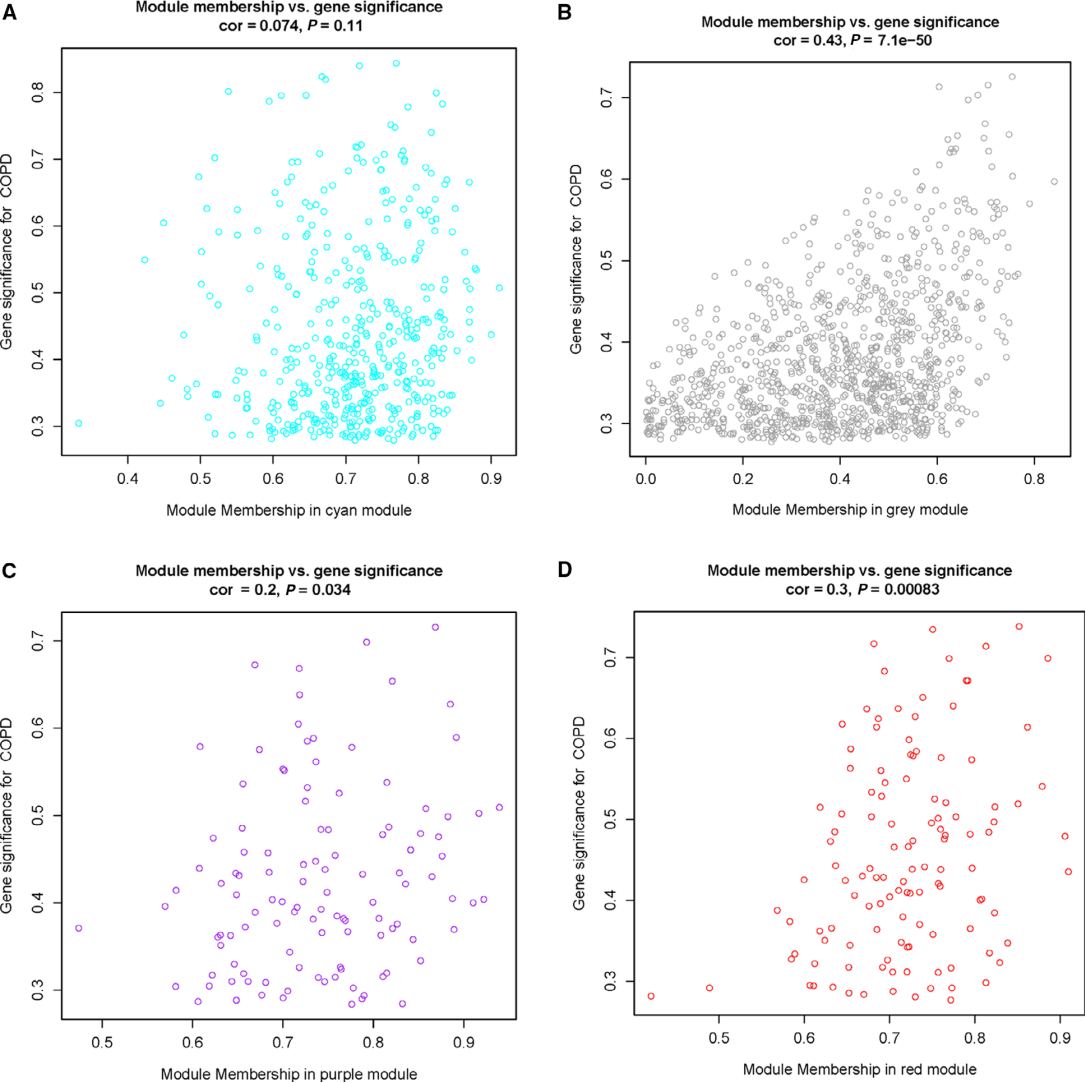
COPD absolute GS versus MM. wgcna calculation of GS to COPD versus MM. In oversimplified terms, MM is a measure of how ‘tight’ genes cluster within the module, or mathematically, how close gene expression is to the module eigenvalue. A gene with high MM and GS identifies Hub genes that are both key components to the underlying BP and highly associated with the trait of interest. The GS for COPD was plotted. (A) Cyan represents MM. (B) Grey represents MM. (C) Purple represents MM. (D) Red represents MM.

Four of these nine gene modules, in Cyan, Purple, Red and Grey, attracted our attention in that 14 Hub genes were identified as DEGs from the PPI analysis, including *CX3CR1*,* PPBP*,* PTGS2*,* FPR1*,* FPR2*,* S100A12*,* ARG1*,* EGR1*,* CD163*,* VCAM1*,* FGG*,* ORM1*,* S100A8* and *S100A9*. We calculated gene significance (GS) versus each MM. We found that the 14 Hub genes were also either positively or negatively associated with COPD (Table 6). *CX3CR1*,* PPBP*,* PTGS2*,* VCAM1*,* S100A12*,* ARG1*,* EGR1*,* CD163*,* S100A8* and *S100A9* were significantly associated with each MM, whereas *FPR1*,* FPR2* and *ORM1* were correlated with each MM except Red MM, and FGG was correlated to each MM except the Purple MM. In addition, we found that the Purple (*CX3CR1*), Red (*EGR1*,* VCAM1* and *PTGS2*) and Grey (*ARG1*,* FGG*, and *PPBP*) genes most significantly correlated with GS for COPD were also the important MM elements (Fig. 11).

**Table 6:** Fourteen Hub genes positively or negatively associated with COPD and each MM. *P*,* P*‐value for COPD or each MM

Gene	Located module	GS.COPD	*P* GS.COPD	*P*.MM Cyan	*P*.MM Grey	*P*.MM Purple	*P*.MM Red
*CD163*	Cyan	−0.739974023	1.33E−15	4.23E−21	8.88E−10	0.00249	2.57E−5
*FPR1*	Cyan	−0.662223538	9.25E−12	7.92E−23	5.84E−7	6.61E−5	0.07339
*FPR2*	Cyan	−0.63983063	7.43E−11	1.37E−21	4.51E−7	6.73E−5	0.07508
*ORM1*	Cyan	−0.459721444	1.23E−5	3.81E−20	0.00014	0.00196	0.70155
*S100A12*	Cyan	−0.721577872	1.41E−14	1.50E−14	2.82E−7	4.13E−5	0.00278
*S100A8*	Cyan	−0.70598989	9.04E−14	6.83E−18	4.01E−7	0.00026	0.01347
*S100A9*	Cyan	−0.623468037	3.07E−10	7.84E−20	4.68E−6	0.00049	0.04290
*ARG1*	Grey	−0.632480057	1.42E−10	0.02374	2.47E−10	0.00054	2.19E−9
*FGG*	Grey	0.304097261	0.00519	0.00646	0.005969	0.2425	3.39E−8
*PPBP*	Grey	−0.520314885	4.61E−7	5.44E−6	0.001842	0.0388	0.01072
*CX3CR1*	Purple	0.553212675	5.84E−8	0.00133	9.38E−10	1.81E−13	4.46E−5
*EGR1*	Red	0.549754835	7.33E−8	0.00127	2.09E−6	0.03903	1.80E−14
*PTGS2*	Red	0.698684808	2.07E−13	0.00341	2.42E−11	4.99E−5	1.10E−28
*VCAM1*	Red	0.7344482490	2.75E−15	0.00947	2.66E−12	5.70E−13	3.14E−16

## Discussion

In this study, we performed an integrated analysis on the gene expression profiles from lung tissues with or without COPD, aiming to identify the DEGs and related key signaling pathways for the disease. We identified 139 DEGs, including 62 up‐regulated genes and 77 down‐regulated genes. In addition, GO analysis showed that the 139 DEGs associated with COPD were involved in 60 BPs, 16 MFs and 12 CCs. Among these categories, the most important BPs included inflammatory response, immune response and response to lipopolysaccharide; the most important MFs included receptor activity and RAGE receptor binding; and the most important CCs included extracellular region and space, integral component of plasma membrane, plasma membrane and external side of plasma membrane. This finding accords with the knowledge that COPD is characterized by chronic inflammation in the lung and airways 28, 29; immune response mediates the development of COPD caused by the harmful stimuli 30, 31, 32; lipopolysaccharide may lead to increased airway and systemic inflammation, and contribute to the progressive deterioration of lung function 33, 34; and RAGE is a ‘driving force’ for cigarette smoke (CS)‐induced airway inflammation in COPD 35.

KEGG pathway analysis indicated that 48 pathways corresponded to these DEGs associated with COPD. Two pathways including hematopoietic cell lineage and cytokine‐cytokine receptor interaction were most important. This finding is in line with the results from previous studies 18, 32.

The PPI network of proteins encoded by DEGs identified 14 Hub DEGs associated with COPD, including *CX3CR1*,* PPBP*,* PTGS2*,* FPR1*,* FPR2*,* VCAM1*,* S100A12*,* ARG1*,* EGR1*,* CD163*,* FGG*,* ORM1*,* S100A8* and *S100A9*. All of these Hub genes were involved in the most important two BPs, two MFs or five CCs revealed by GO analysis, and were mainly implicated in multiple pathways identified by KEGG analysis in this study. Those results indicate that these Hub DEGs are involved in the development and progression of COPD by playing important biological roles in multiple signaling pathways.

Using wgcna on the merged expression profile from two cohorts of lung tissues with COPD and healthy controls, we identified a set of gene signatures based on the 14 Hub genes. The increased expression of *CX3CR1*,* FGG*,* EGR1*,* VCAM1* and *PTGS2* is positively associated with COPD, and the underexpression of *PPBP*,* FPR1*,* FPR2*,* S100A12*,* ARG1*,* CD163*,* ORM1*,* S100A8* and *S100A9* is negatively associated with COPD.

*CX3CR1* plays an important role in the development of chronic inflammatory lung diseases, such as COPD and emphysema, by contributing to structural destruction and remodeling. Chemoattractant inflammatory cells releasing *CX3CR1*, such as CD8^−^/CD4, dendritic cells, γδ T lymphocytes, natural killer cells and monocytes/macrophages, may lead to mononuclear cell accumulation in the parenchyma and lung vessel walls, release mediators to induce injury, stimulate proliferation and chemoattractant inflammatory cells 36. In addition, *CX3CR1* ^+^ mononuclear phagocytes may induce an innate immune response to CS via producing interleukin‐6 and tumor necrosis factor‐α, and contribute to emphysema 37.

*PTGS2* (*COX‐2*), an important mediator of inflammation, was shown to be involved in inflammation response and associated with COPD pathogenesis 38, 39, 40, 41. The decreased activity of *PTGS2* may protect smokers against the development of COPD 40. Furthermore, *FPR1* and *FPR2* were reported to be involved in recruitment and activation of inflammatory cells induced by CS, and play important roles in lung inflammation, injury and the pathogenesis of COPD 42, 43, 44, 45.

*S100A8*,* S100A9*, and *S100A12* might induce neutrophil and monocyte chemotaxis, adhesion to fibrinogen and diapedesis, and neutrophil migration to inflammatory sites 46, 47, and have been identified as key biomarkers in inflammatory diseases including COPD and neutrophil‐dominated infections 35, 48, 49. The mRNA levels of *S100A8*,* S100A9* and *S100A12* may be regulated by RAGE, which was shown to contribute to CS‐induced airway inflammation in COPD 35. This is consistent with our result in this study that the RAGE pathway including *S100A8*,* S100A9* and *S100A12* is important in the development of COPD.

*EGR1* is an autophagy regulator gene that plays important roles in cellular homeostasis, airway remodeling and control of inflammatory immune response; it is also a significant risk factor for susceptibility to COPD 50, 51, 52. *EGR1* may be induced by CS and involved in proinflammatory mechanisms that are likely associated with the development of COPD 51, whereas Egr‐1^−/−^ mice were observed to resist CS‐induced autophagy, apoptosis and emphysema 53. These findings exhibit a critical role for *EGR1* in CS‐induced inflammatory immune response and COPD, and effective inhibition of *EGR1* may attenuate airway remodeling and inflammation associated with the pathology of COPD.

*CD163*, a carefully regulated component of the innate immune response to infection and a macrophage receptor for bacteria, was shown to play important roles in functional pulmonary defense elements and the inflammatory immune response of the respiratory system 54, 55. Overexpression of *CD163* on lung alveolar macrophages may be implicated in the pathogenesis of COPD and poor lung function 56.

*ARG1* was shown to contribute to asthma pathogenesis by inhibiting nitric oxide production, modulating fibrosis, regulating arginine metabolism and inhibiting T cell proliferation, and it involves the initiation of adaptive T helper 2 cell‐mediated allergic lung inflammation by regulating group 2 innate lymphoid cells 57, 58, 59, 60, whereas *ARG1* ablation in the lung may enhance peripheral lung function but have little effect on airway inflammation 61. The role of *ARG1* in COPD needs to be studied in the future.

*ORM1* appears to function in regulating the activity of the immune system during the acute‐phase reaction and has been identified as an acute exacerbation of COPD‐specific immunomodulatory mediator 62. *PPBP* serves as a potent neutrophil chemoattractant and activator, and its elevated expression in the bronchial mucosa might be involved in the pathogenesis of COPD 63, 64. In addition, *VCAM1* was shown to express in endothelial cells of atopic asthma cases, but not COPD cases 65, and present an association with lung function 66. *FGG* was found to be involved in blast lung injury resistance via promoting tissue‐protective adenosine signaling 67. In the lung tissues of COPD cases, its mRNA expression was reported to correlate with the burden of particulate matter in total lung and lung parenchyma 68.

In conclusion, we have identified 139 candidate DEGs associated with the progression of COPD. The results from bioinformatic analysis are in agreement with those from previous cell and animal models and human studies. Our results showed that nine Hub genes, *CX3CR1*,* PTGS2*,* FPR1*,* FPR2*,* S100A12*,* EGR1*,* CD163*,* S100A8* and *S100A9*, potentially mediated inflammation and injury of the lung, and play critical roles in the pathogenesis of COPD. The roles of five Hub genes, including *PPBP*,* ARG1*,* FGG*,* VCAM1* and *ORM1*, identified to be associated with COPD in this study need to be confirmed in the future. These findings could improve our understanding of the underlying molecular mechanisms of COPD and provide us with insights for drug target discovery for the disease.

## Conflict of interest

The authors declare no conflict of interest.

## Author contributions

XK, FY and QW conceived and designed the project. XH and YL acquired the data. XG and ZZ analyzed and interpreted the data. XH and YL wrote the paper.
